# Performance Evaluation of the VIDAS^®^ Measles IgG Assay and Its Diagnostic Value for Measuring IgG Antibody Avidity in Measles Virus Infection

**DOI:** 10.3390/v8080234

**Published:** 2016-08-20

**Authors:** Julia Dina, Christian Creveuil, Stephanie Gouarin, Florent Viron, Amelie Hebert, Francois Freymuth, Astrid Vabret

**Affiliations:** 1 Department of Virology, CHU de Caen, F-14000 Caen, France; gouarin-s@chu-caen.fr (S.G.); viron-f@chu-caen.fr (F.V.); hebert-am@chu-caen.fr (A.H.); freymuth.francois@wanadoo.fr (F.F.); vabret-a@chu-caen.fr (A.V.); 2 Normandy University, Faculty of Medicine, UNICAEN, EA4655, F-14000 Caen, France; 3 National Reference Center (NRC) for Measles and Paramyxoviridae Respiratory Viruses, F-14000 Caen, France; 4 Department of Biostatistics and Clinical Research, CHU de Caen, F-14000 Caen, France; creveuil-cr@chu-caen.fr

**Keywords:** measles virus, measles diagnosis, measles avidity

## Abstract

The objective of this study is primarily to compare the performance of the VIDAS^®^ Measles immunoglobulin (Ig)G assay to that of two other serological assays using an immunoassay technique, Enzygnost^®^ Anti-measles Virus/IgG (Siemens) and Measles IgG CAPTURE EIA^®^ (Microimmune). The sensitivity and the agreement of the VIDAS^®^ Measles IgG assay compared to the Enzygnost^®^ Anti-measles Virus/IgG assay and the Measles IgG CAPTURE EIA^®^ assay are 100%, 97.2% and 99.0%, 98.4%, respectively. The very low number of negative sera for IgG antibodies does not allow calculation of specificity. As a secondary objective, we have evaluated the ability of the VIDAS^®^ Measles IgG assay to measure anti-measles virus IgG antibody avidity with the help of the VIDAS^®^ CMV IgG Avidity reagent, using 76 sera from subjects with measles and 238 other sera. Different groups of populations were analyzed. In the primary infection measles group, the mean IgG avidity index was 0.16 (range of 0.07 to 0.93) compared to 0.79 (range of 0.25 to 1) in the serum group positive for IgG antibodies and negative for IgM. These data allow to define a weak anti-measles virus IgG antibody avidity as an avidity index (AI) < 0.3 and a strong avidity as an AI > 0.6. The VIDAS^®^ Measles IgG assay has a performance equivalent to that of other available products. Its use, individual and quick, is well adapted to testing for anti-measles immunity in exposed subjects.

## 1. Introduction

Measles is one of the most contagious infectious diseases, caused by the measles virus (MeV), a single-stranded RNA virus of the *Paramyxoviridae* family. Although measles is primarily considered to be a childhood disease, it can affect people of all ages. The introduction of a live measles vaccine has been associated with a dramatic reduction in measles. However, despite the vaccination programs adopted by many developed countries and advancements towards the goal of measles elimination, outbreaks continue to occur in Europe and more recently in the USA [1,2,3,4,5,6,7,8]. 

Lifelong immunity is generally reported after a wild-type measles infection, while in vaccinated people a waning immunity has been reported in correlation with lower levels or more rapid decrease of measles specific antibodies [9,10,11]. Asymptomatic reinfections or disease were observed in subjects where the immune system was challenged by 2-vaccine doses, probably because of an inadequate response. The proportion of vaccinated 20 to 24-year-old patients in the last French outbreak was 4.8% [12]. Furthermore, occasional spread from unvaccinated patients with measles to 2-dose vaccine recipients has recently been observed [13,14,15].

The risk of measles complications makes it important to rapidly detect the immunological status of the contact patients suffering from measles in order to identify those not immunized and exposed, and to vaccinate when necessary. In previously immunized and inadequately immunized individuals, laboratory confirmation of acute measles infection is a challenge. IgM response may be absent or short-lived, and RNA detection very limited. In this case, an IgG avidity test could help clinicians to distinguish between primary and secondary measles infection. The avidity test is a technique that enables weak avidity antibodies, produced at the early stages of a primary infection, to be differentiated from high avidity antibodies, which are characteristic of a reinfection.

Sensitive and specific commercial kits are widely available for the detection and the quantification of measles antibodies, and enzyme immunosorbent assay (EIA) is the most widely used test format. Nevertheless, others kits where adapted to evaluate the avidity of IgG measles antibodies [16,17]. 

The VIDAS^®^ Measles IgG assay is an individual, qualitative, and automated assay, allowing for the detection of anti-measles virus IgG using the enzyme-linked fluorescent assay (ELFA) method [18]. A commercialized IgG measles avidity test is not available, but a very robust VIDAS^®^ CMV avidity test (bioMérieux, Marcy-l’Etoile, France) exists and is routinely used to determine the date of infection in case of suspicion of a cytomegalovirus (CMV) infection. 

The objective of this study was, first, to compare the performance of the VIDAS^®^ Measles IgG assay to those of two other serological assays, Enzygnost^®^ Anti-measles Virus/IgG (Siemens, Marburg, Germany), and Measles IgG capture EIA^®^ (Microimmune, Brentford, UK) and, second, to study whether the VIDAS^®^ Measles IgG assay, used with the VIDAS^®^ CMV IgG Avidity reagent (bioMérieux) to determine the avidity of anti-measles virus IgG antibody, yields consistent results on serum with recent or past immunity profiles.

## 2. Material and Methods

### 2.1. Presentation of the Kits Used in This Study

The VIDAS^®^ Measles IgG test (bioMérieux) is a qualitative, individual, and automated assay allowing a rapid analysis of serum samples to confirm the presence or absence of anti-measles IgG. This assay combines an immunoenzymatic sandwich technique with an enzyme-linked fluorescent assay (ELFA) on a specific instrument. A pipette tip-like disposable device coated with the antigen, constitutes the solid phase and serves as the pipettor. All of the other reagents are presented in a 10-well foil-sealed strip. The results are expressed in assay values: negative, equivocal, and positive for the assay values <0.50, ≥0.50 to <0.70, and ≥0.70 respectively. 

The Enzygnost^®^ Anti-measles Virus/IgG assay (Siemens, Marburg, Germany) is an indirect enzyme-linked immunosorbent assay (ELISA) assay on a microtitration plate coated with inactivated measles antigen, which is read on an ELISA processor. The calculation of the results takes into account the absorption of the cellular system used to produce the virus. Results are expressed in mean absorbance values (ΔA), negative, equivocal, and positive for the assay values <0.100, ≥0.100 to ≤0.200, and >0.200, respectively. A quantitative result can be obtained for samples where the mean absorbance was superior to the limit value (0.100), calculated using the α method and expressed in mIU/mL. 

In the Microimmune Measles IgG capture EIA^®^ assay (Microimmune), a serum is added to anti-human IgG coated microtitre wells. IgG in the specimens binds to the wells, and, recombinant measles nucleoprotein (rMVN) antigen is added after washing. Measles specific IgG in the sample, if present, binds to the rMVN. Then, a monoclonal antibody anti-rMVN conjugated to horseradish peroxidase (HP) is added. The presence of specific IgG is revealed by a color change after adding the HP substrate. The color change and intensity are monitored using a spectrophotometric plate reader. Negative, equivocal and positive results are expressed in optical density values (OD) established from threshold values defined for each series of assays (from 0.098 to 0.135).

The avidity test is based on the functional binding or avidity of IgG antibodies increasing progressively over time after immunization, known as maturation of the humoral immune response. Presence of low avidity IgG antibodies may indicate a primary infection whereas high percentage of high avidity IgG antibodies may indicate a recurrent infection. To determine anti-measles IgG avidity, the urea reagent of the VIDAS^®^ CMV IgG Avidity assay was used with the solid phase reagents and strips from the VIDAS^®^ Measles IgG kit. Each serum was treated using two VIDAS^®^ Measles IgG tests. One test served as the reference. In the other, the wash buffer in well 4 of the strip was replaced with the buffer containing 6 M urea from the VIDAS^®^ CMV IgG Avidity kit. The avidity index (AI) was determined by calculating the ratio between the relative fluorescent values (RFV) obtained with the reference strip and the RFV obtained with the strip containing urea. 

All the techniques were performed following manufacturers’ instructions except for the above-mentioned measles avidity assay.

### 2.2. Selection of the Sera

In order to constitute a representative panel, three different groups of sera (A, B and C) comprising a total of 321 human sera were selected for this study. The sera were collected from January 2011 to December 2012. Group A included 76 sera from subjects with laboratory confirmed measles. For these patients, the clinical symptoms suggested measles and were confirmed by the presence of specific IgM and IgG antibodies in Enzygnost^®^ Anti-measles Virus/IgG and Enzygnost^®^ Anti-measles Virus/IgM assays. Group B included 125 sera from two types of patients with a suspicion of measles based on clinical symptoms: patients for which the serological tests performed in different laboratories using different tests did not confirm the measles infection, and patients having measles in their family circle and in which a serology was performed to establish if these people were immunized against measles or not. Group C included 120 sera, originating from blood donors, subjects without of any suspicion of measles infection or contact cases. The three serum groups were formed to have equivalent mean age.

All serum samples were conserved at −20 °C. Analyses by the different assays were performed in parallel for each sample. 

For the avidity study, 314 sera with a positive result for IgG measles antibodies (mean absorbance value ΔA > 0.2) for the Enzygnost^®^ Anti-measles Virus/IgG assay were selected (76, 125 and 113 of the sera from groups A, B, and C, respectively). These sera were tested using the protocol described above. To assess the intra-assay precision of this protocol, two serum samples—with low and high MeV-IgG avidity—were tested three times each with the VIDAS^®^ instrument. 

### 2.3. International Standard

An international standard (National Institute for Biological Standards and Control (NIBSC) code: 97/648) was used to define the threshold concentration of neutralizing antibodies corresponding to the positive result in the VIDAS^®^ Measles IgG and Enzygnost^®^ Anti-measles Virus/IgG assays. This standard contains 3000 mIU/mL of measles virus neutralizing antibodies and is prepared using the plaque reduction technique. Dilutions of this standard were made in a pool of sera negative for anti-measles IgG and tested three times with each reagent. For each reagent, the results were graphically analyzed by representing the average of the three assay values as a function of the international standard concentration. 

### 2.4. Statistical Analysis

Ninety-five percent exact binomial confidence intervals (95% CI) were computed for sensitivity, specificity and agreement measures. Mean avidity indexes were compared between the three groups using an analysis of variance. Statistical significance was defined as *p* < 0.05. IBM SPSS software, version 22, was used for the analysis. 

A receiver operating characteristic (ROC) curve analysis was used and the area under the curve (AUC) was calculated to evaluate diagnosis ability of the avidity index to discriminate group A from group C, and group B from group C, finding the optimal cut-off values along with sensitivity and specificity.

## 3. Results

### 3.1. Population Characteristics

Group A included four children less than 10 years old and 72 adults: mean age 33.1 years (17 to 83 years). Group B included seven children less than 10 years old and 118 adults: mean age 32.5 years (17 to 78 years); Group C comprised 120 adults: mean age 35.5 years (18 to 61 years). The female to male ratio was 1.1, 2.4, and 1.0 in the three groups A, B, and C, respectively.

### 3.2. Relative Performance of the VIDAS^®^ Assay

Out of the 321 sera analyzed by the VIDAS^®^ Measles IgG and Enzygnost^®^ Anti-measles Virus/IgG assays reagents, 309 (96.3%) were found to be positive by the VIDAS^®^ Measles IgG assay and 304 (94.7%) by the Enzygnost^®^ Anti-measles Virus/IgG assay (Table 1). The five (1.6%) sera found to be equivocal by the VIDAS^®^ Measles IgG assay and the 10 (3.1%) sera equivocal by Enzygnost^®^ Anti-measles Virus/IgG were considered as negative in the performance analysis. Whenever viral serology was performed, an equivocal result led to a confirmatory test on a second serum sampled a few days later after the first serum. The relative sensitivity and the agreement of the VIDAS^®^ Measles IgG assay compared to the Enzygnost^®^ Anti-measles Virus/IgG assay were 100% (95% CI: 98.8%; 100%) and 97.2% (95% CI: 94.7%; 98.7%), respectively. All 76 sera from Group A (clinical measles) were positive for anti-measles IgG using both the VIDAS^®^ Measles IgG and Enzygnost^®^ Anti-measles Virus/IgG assays.

All 321 sera were also analyzed in parallel by the VIDAS^®^ Measles IgG and Microimmune Measles IgG capture EIA^®^ assays. In total, 309 (96.3%) were found to be positive by the VIDAS^®^ Measles IgG assay and 311 (96.9%) by the Microimmune Measles IgG capture EIA^®^ assay (Table 2). Five sera (1.6%) were found to be equivocal by VIDAS^®^ Measles IgG assay and three (0.9%) sera were found to be equivocal by Microimmune Measles IgG, and these results were considered as negative in the performance analysis of the test. The relative sensitivity and the agreement of the VIDAS^®^ Measles IgG assay compared to the Microimmune Measles IgG capture EIA^®^ assay were 99.0% (95% CI: 97.2%; 99.8%) and 98.4% (95% CI: 96.4%; 99.5%), respectively. 

### 3.3. Detection Threshold

**Figure 1:**
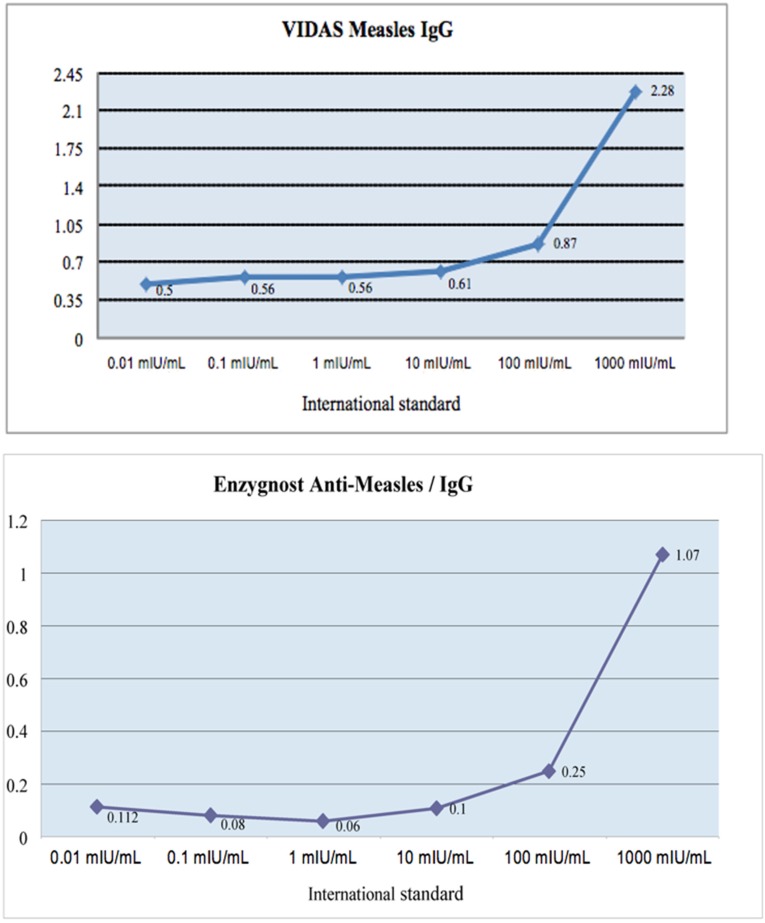
Positive result thresholds for VIDAS^®^ Measles IgG and Enzygnost^®^ Anti-measles Virus/IgG assay. (**a**) VIDAS^®^ Measles IgG threshold; (**b**) Enzygnost^®^ Anti-measles Virus/IgG assay thresholds.

The positive result threshold for both tests, VIDAS^®^ Measles IgG (>0.7) and Enzygnost^®^ Anti-measles Virus/IgG (>0.2) assays corresponds to the same international standard concentration, i.e., 100–150 mIU/mL of antibodies neutralizing the Edmonston virus strain of measles (Figure 1). 

### 3.4. Avidity Index for Anti-Measles Virus IgG

**Figure 2:**
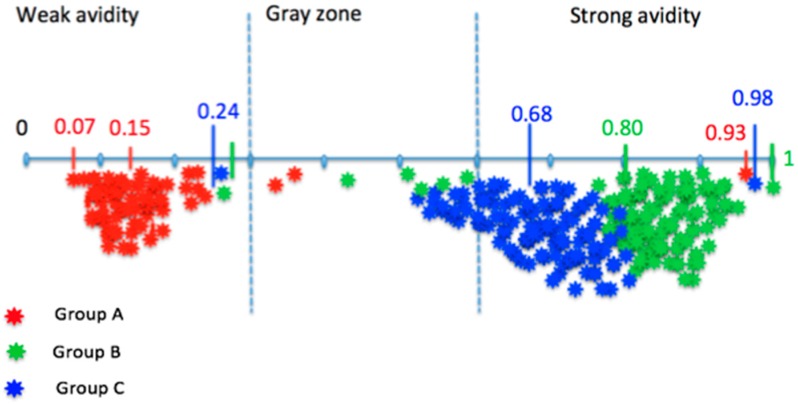
Schema of the distribution of the avidity index (AI) for anti-measles virus IgG results.

In Group A, including 76 primary measles infections, the mean avidity index for IgG was 0.16 (range of 0.07 to 0.93). Seventy-three sera had an AI ≤ 0.30; two sera had an avidity range from 0.30 to 0.60 and 1 serum had an AI > 0.60. Group B included 125 sera that were IgG positive and IgM negative. The mean AI for IgG was 0.79 (range of 0.25 to 1). Group C, from 113 blood donors, presented a mean AI of 0.67 (range of 0.24 to 0.98). One serum had an AI < 0.30. Mean avidity indexes were significantly different between the three groups (*p* < 0.001). The IgM antibodies were negative by the Measles IgM capture EIA^®^ Microimmune assay (Figure 2).

These data define a weak anti-measles virus IgG avidity for an AI ≤ 0.3, a strong avidity for an AI > 0.6, and a gray zone for AI values >0.3 and ≤0.6. The sensitivity of the avidity assay can be established on the percentage of sera with weak avidity in Group A clinical measles with positive IgM: 96.1% (73/76) (95% CI: 88.9%; 99.2%). Out of the three sera having an AI > 0.30, two had an equivocal AI, from 0.33 to 0.49, and one serum had strong avidity IgG, AI = 0.92. The specificity corresponds to the percentage of sera with strong avidity over the total samples in Groups B and C, where measles was not confirmed by biology, and it was 85.3% (203/238) (95% CI: 80.1%; 89.5%). The serums from Groups B and C did not have IgM specifically detectable by the Enzygnost^®^ Anti-measles Virus/IgM assay. Nevertheless, group B included eight positives sera and two equivocal sera for IgM by the Measles IgM capture EIA^®^ Microimmune assay at values higher than the threshold. The mean titer for IgG antibodies of sera belonging to groups B and C was 8168.2 mIU/mL, and only one serum had a titer lower than 1000 mIU/mL. The AI of all these sera was >0.70. 

**Figure 3:**
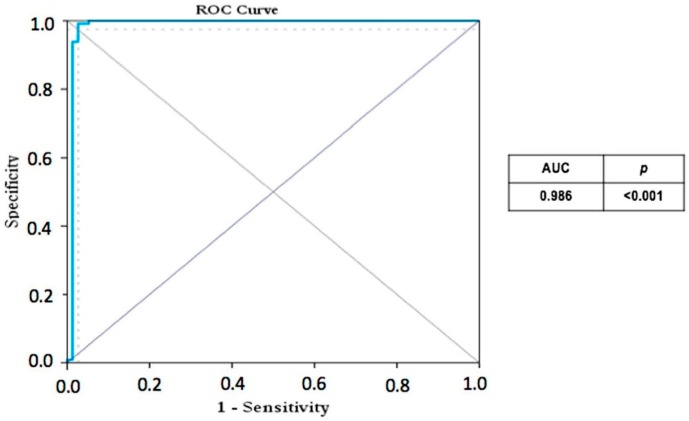
Receiver operating characteristic ROC curve where group A versus group C was analyzed.

The ROC analyses of the avidity test show a sensibility of 94.7%, CI 95% (89.7; 99.7) and a specificity of 99.1%, CI 95% (97.3; 100.0) for the best threshold who maximize the sensitivity and the specificity, 0.24. The AUC for the avidity index was 0.986 (CI 95%, from 0.96 to 1.00) (*p* < 0.001) (Figure 3).

**Figure 4:**
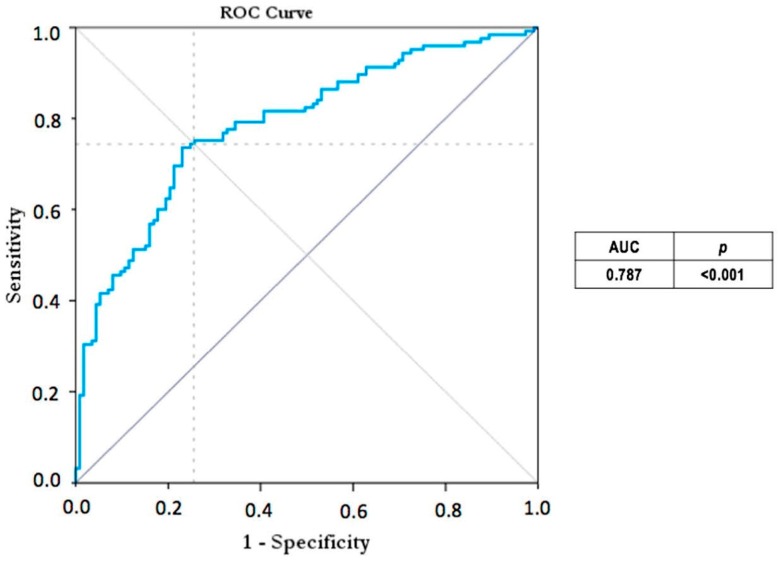
ROC curve where group B versus group C was analyzed.

When groups B and C were compared, the best threshold that maximized the sensibility and the sensitivity was 0.74. This value shows a sensibility of 75.2%, CI 95% (67.6; 82.7) and a specificity of 74.3%, CI 95% (66.2; 82.3). For this score the AUC was 0.787 (CI 95%, from 0.73 to 0.84) (Figure 4).

## 4. Discussion

A successful immunization program must be able to quickly identify system weakness and target susceptible subjects. A wild-type measles infection or a 2-dose measles vaccination is correlated with lifelong immunity. Disease or vaccination histories are based on maternity reports, clinical records, or a vaccination notebook. Sometimes this information is not available or it is subject to error and bias. The rapid detection of the immunological status of contact patients with measles is important in some clinical situations such as pregnancies, immunosuppressed patients and patients with other underlying medical conditions, and families with children too young to be vaccinated.

This study evaluated the performance of the VIDAS^®^ Measles IgG assay, a qualitative, individual, and automated assay allowing for a rapid analysis of the serum samples compared to those of two other serological assays, Enzygnost^®^ Anti-measles Virus/IgG (Siemens), and Measles IgG capture EIA^®^ (Microimmune). Measles IgG capture EIA^®^ (Microimmune) is a test developed for the detection of measles antibodies in saliva and it can also be used for serum samples. The relative sensitivity and the agreement of the VIDAS^®^ Measles IgG assay were 100% and 97.2%, respectively when compared to the Enzygnost^®^ Anti-measles Virus/IgG assay and 99% and 98.4%, respectively when compared to the Microimmune Measles IgG capture EIA^®^ assay. The very small number (eight to 11) of negative sera for anti-measles virus IgG antibodies, does not allow to interpret the relative specificity percentages for the VIDAS^®^ Measles IgG assay compared to the two other assays. Recently, González-Escalada compared the Enzygnost^®^ EIA test to the VIDAS Measles IgG test. In this study the sensitivity reported for VIDAS^®^ test was 98% and its specificity was 78.1% [19]. 

Although the significant increase in specific IgG antibodies that accompanies measles infection can be confirmed using measles IgG assay, time-to-result is too long and, in practice, a confirmed diagnosis relies on detection of the viral RNA by reverse transcription PCR (RT-PCR) in the saliva or nasopharynx swabs, and/or on detection of both specific IgG and IgM in the serum or saliva of the patient [20].

The VIDAS^®^ Measles IgG assay is an individual assay, that is easy-to-use, rapid and which can be used, for example, on people in contact with a patient infected by measles so as to identify who is not immunized and exposed, and to vaccinate when necessary. Protection against measles generally means that the subject will not have the disease again; it does not guarantee that a subject cannot be infected again. It has been shown that measles virus reinfections or measles disease were observed in subjects whose immune system had been strained by the wild virus or the vaccine [21,22,23]. This was verified during the recent measles epidemic in France from 2008 to 2011. The proportion of subjects who had measles among those immunized with one dose of Measles-Mumps-Rubella (MMR) vaccine was 10.7%, while among subjects who received two doses of the vaccine it was 4.8%, and 85.6% among non-vaccinated subjects [24].

In comparison to protection against measles disease, protection against reinfection by the measles virus requires a higher level of neutralizing antibodies. The study by Chen et al. shows that none of the seven children with a neutralizing antibody level higher than 1.025 mIU/mL contracted an asymptomatic infection [25]. In our study, it should be observed that only six of the 125 sera (4.8%) from the subjects considered as immunized against measles (Group B) had an antibody level higher than 1.000 mIU/mL. The threshold at which an individual can be considered as protected against infection by the measles virus is not defined in the literature. It should probably be set at above 1.000 mIU/mL.

Measuring the antibodies to determine who has seroconverted one month after immunization could be logistically difficult and expensive. Alternatively, avidity testing allows the measurement of the functional affinity of anti-viral IgG concomitant with measles laboratory diagnosis in individuals whose immunization has failed. In the second part of our study, we assessed the possibility of measuring anti-measles virus IgG avidity with a VIDAS^®^ Measles IgG assay, completed with VIDAS^®^ CMV IgG Avidity assay reagents. The measurement of anti-measles virus IgG antibody avidity was developed in order to differentiate primary infections from re-infections [26], and also to characterize measles vaccine failure [16,27]. A weak IgG avidity is compatible with a recent infection. This may be useful given the variable specificity of IgM detection observed in available EIA Kits. One Canadian study comparing five immunoassays did not show significant differences between indirect and immunocapture ELISA assays. On the other hand, the positive result rates for IgM vary from 57% to 80% between kits for sera taken during the acute phase, or on average within the four days after the beginning of the symptoms [28]. Moreover, during the course of a primary infection by measles, specific IgM antibody titers decrease after the fourth or fifth weeks, leading to equivocal results; moreover, viral RNA is no longer detectable in the saliva. In this case, the observation of weak IgG avidity could confirm the diagnosis of primary infection [24]. 

The avidity assay is also important for characterizing measles in populations vaccinated by the MMR vaccine. In this case, measles disease corresponds either to a failure of primary vaccination (weak IgG avidity), or to that of the booster (strong IgG avidity) [16]. In Group A, one of the 76 sera presented a strong IgG avidity (AI = 0.93). The sera belonged to a 32-year-old subject who, in the middle of the epidemic in France in 2011, presented typical measles with very elevated anti-measles virus IgM and IgG antibodies. The strong IgG avidity suggested a secondary infection in a subject who had probably received one dose of vaccine in his childhood. However, this information could not be verified. Likewise, eight sera from Group B, initially reported as IgM negative, were found positive by the Measles IgM CAPTURE EIA^®^ assay and had an AI > 0.77. These sera came from young adults with a mean age of 32.5 years. These were cases of contracted measles in which the diagnosis was not initially confirmed by serology, and could be attributed to a false negative. 

With the aim of measuring IgG avidity on a modified VIDAS^®^ Measles IgG assay, in the present study we have defined thresholds by defining weak, i.e., <0.3, and strong anti-measles virus IgG antibody avidity, i.e., >0.6. These thresholds correspond to those published in the literature with other tests, which vary between <0.3 [16,17,27] and <0.45 [29] for weak, and between 0.5 [27,29] and 0.7 [16,17] for strong IgG avidity. These thresholds where confirmed by ROC analyses. Further validation of these thresholds on another cohort of samples is recommended.

## 5. Conclusion

The performance of the VIDAS^®^ Measles IgG assay was tested in populations with different immunological status, patients with laboratory confirmed measles, patients with measles symptoms which were not confirmed by serology and blood donors with IgG antibodies. In all these situations the performance of VIDAS^®^ Measles IgG assay showed over 97% agreement with two other commercial immunoassays. These characteristics, together with the individual/single-dose format and rapidity of the test, make the VIDAS^®^ Measles IgG assay suitable for testing exposed subjects for measles immunity. This study also shows that a modified protocol of the VIDAS^®^ Measles IgG assay combined with VIDAS^®^ CMV avidity test reagents allows for the determination of IgG measles antibody avidity. The results of detection of IgG antibody avidity are particularly useful for the serological diagnosis of measles infection when performed late, in a context of secondary infection, in pregnancy, and in patients with underlying pathologies. 

## Figures and Tables

**Table 1:** Comparison of the VIDAS^®^ Measles IgG assay and the Enzygnost^®^ Anti-measles Virus/IgG assay.

		Enzygnost^®^ Measles IgG			Total
		Negatives	Equivocal	Positives	
**VIDAS^®^ Measles IgG**	**Negatives**	5	2	0	7
	**Equivocal**	2	3	0	5
	**Positives**	0	5	304	309
**Total**		7	10	304	321

**Table 2:** Comparison of the VIDAS^®^ Measles IgG assay and the Measles IgG capture EIA^®^ Microimmune assay.

		Measles IgG Capture EIA^®^ Microimmune			Total
		Negatives	Equivocal	Positives	
**VIDAS^®^ Measles IgG**	**Negatives**	6	0	1	7
	**Equivocal**	1	2	2	5
	**Positives**	0	1	308	309
**Total**		7	3	311	321
